# Piloting an Augmented Reality Life Review Experience to Promote Mental Health Outcomes in Aging Asian American Women

**DOI:** 10.1093/geroni/igab046.3138

**Published:** 2021-12-17

**Authors:** Sarah Hwang, Jazlyn Armendariz, Jeremy Argueta, Veronica Fruiht, Thomas Chan

**Affiliations:** 1 California State University Northridge, Northridge, California, United States; 2 Dominican University of California, Dominican University of California, California, United States

## Abstract

Asian-American older women report the highest prevalence of suicidal ideations and rates of completed suicide compared to other racial groups. Ironically, Asian-American communities report disproportionately low rates of formal mental health utilization—this may be attributed to the lack of culturally-relevant services and negatively ingrained perceptions of mental health aid. One potential solution that has not been widely investigated is the use of technology to help older Asian-American women engage in mental health interventions. This study leverages innovations in augmented reality (AR) technology (i.e., overlaying of digital holograms onto the real world) to create a life review intervention aimed at promoting mental health well-being. The application, Tell-Being, is a personalized holographic life review experience that facilitates older adults to foster a sense of coherence and wholeness within their lives. Pilot data collection was amassed from four aging Asian-American female participants averaging 51.3 (SD=8.61) years of age. Initial pre/post analyses showcased mean differences that trend towards a higher presence of emotion regulation from pre-test (M=4.88, SD=1.08) to post-test (M=5.21, SD=1.17). Although data collection was prematurely halted due to COVID-19, results trended in promising directions. The technological innovations and findings from this study may lead to promising novel avenues to address barriers for older Asian-American women in seeking mental health assessment and treatment in a “new normal” world.

